# Can asthma control be improved by understanding the patient's perspective?

**DOI:** 10.1186/1471-2466-7-8

**Published:** 2007-05-22

**Authors:** Rob Horne, David Price, Jen Cleland, Rui Costa, Donna Covey, Kevin Gruffydd-Jones, John Haughney, Svein Hoegh Henrichsen, Alan Kaplan, Arnulf Langhammer, Anders Østrem, Mike Thomas, Thys van der Molen, J Christian Virchow, Siân Williams

**Affiliations:** 1 Professor of Behavioural Medicine, Centre for Behavioural Medicine, School of Pharmacy University of London, UK; 2 General Practitioner, Norwich, UK; Professor, Department of General Practice and Primary Care, University of Aberdeen, Aberdeen, UK; 3 Department of General Practice and Primary Care, University of Aberdeen, Aberdeen, UK; 4 General Practitioner, Porto, Portugal; 5 Chief Executive, Asthma UK, London, UK; 6 General Practitioner, Box, UK; School for Health, University of Bath, UK; 7 General Practitioner, Glasgow, UK; Department of General Practice and Primary Care, University of Aberdeen, Aberdeen, UK; 8 General Practitioner, Oslo, Norway; 9 General Practitioner, Richmond Hill, Canada; Emergency Department, York Central Hospital, Richmond Hill, Canada; 10 General Practitioner, Steinkjer, Norway; HUNT Research Centre, Norwegian University of Science and Technology, Verdal, Norway; 11 General Practitioner, Oslo, Norway; 12 General Practitioner, Minchinhampton, UK; Department of General Practice and Primary Care, University of Aberdeen, Aberdeen, UK; 13 Professor, Department of General Practice University Medical Centre Groningen, Groningen, Netherlands; 14 Professor, Clinic for Internal Medicine, University of Rostock, Rostock, Germany; 15 Executive Officer, International Primary Care Respiratory Group, London, UK

## Abstract

**Background:**

Clinical trials show that asthma can be controlled in the majority of patients, but poorly controlled asthma still imposes a considerable burden. The level of asthma control achieved reflects the behaviour of both healthcare professionals and patients. A key challenge for healthcare professionals is to help patients to engage in self-management behaviours with optimal adherence to appropriate treatment. These issues are particularly relevant in primary care, where most asthma is managed. An international panel of experts invited by the International Primary Care Respiratory Group considered the evidence and discussed the implications for primary care practice.

**Discussion:**

Causes of poor control

Clinical factors such as exposure to triggers and concomitant rhinitis are important but so are patient behavioural factors. Behaviours such as smoking and nonadherence may reduce the efficacy of treatment and patients' perceptions influence these behaviours. Perceptual barriers to adherence include doubting the need for treatment when symptoms are absent and concerns about potential adverse effects. Under-treatment may also be related to patients' underestimation of the significance of symptoms, and lack of awareness of achievable control.

Implications

Three key implications for healthcare professionals emerged from the debate. First, the need for simple tools to assess asthma control. Two approaches considered were the monitoring of biometric markers of control and questionnaires to record patient-reported outcomes. Second, to understand the reasons for poor control for individual patients, identifying both clinical (e.g. rhinitis) and behavioural factors (e.g. smoking and nonadherence to treatment). Third was the need to incorporate, within asthma review, an assessment of patient perspectives including their goals and aspirations and to elicit their beliefs and concerns about asthma and its treatment. This can be used as a basis for agreement between the healthcare professional and patient on a predefined target regarding asthma control and a treatment plan to achieve this.

**Summary:**

Optimum review of asthma is essential to improve control. A key priority is the development of simple and effective tools for identifying poor control for individual patients coupled with a tailored approach to treatment to enable patients to set and achieve realistic goals for asthma control.

## Background

Asthma is a chronic inflammatory disease of the airways, resulting in widespread but variable airflow obstruction in response to a variety of stimuli[1]. Airflow obstruction is usually reversible, either spontaneously or with treatment, though remodelling may lead to irreversible structural changes.

National and international guidelines clearly state that the aim of asthma management is to achieve and maintain control[1,2]. Controlled asthma is characterised by minimal or no symptoms during the day and at night, no asthma attacks, no emergency visits to physicians or hospitals, minimal need for reliever medications, no limitations on physical activities and exercise, nearly normal lung function and minimal or no side-effects from medication.

With the medical treatments currently available, it is possible to achieve control in the majority of patients with asthma, at least in the artificial setting of a clinical trial[3]. However, in the real world where patients make choices that may reflect conflicting priorities, asthma still imposes a considerable burden on healthcare systems, largely as a result of poor control.

There is evidence, from a 10 year Finish study, that enhancing the delivery of healthcare services, can improve asthma control[4] but, in most countries, poor control remains a significant burden for patients and the healthcare system. An analysis of nine studies conducted in Australia, Canada, France, Sweden, UK and USA showed that around one third of the direct costs of asthma, and three-quarters of the total costs of asthma, were a consequence of uncontrolled disease[5]. In a US study conducted in 1993, the average cost per patient ranged from US$47 for those with controlled disease, to US$7030 for those with uncontrolled symptoms[6]. A survey in the UK found that the annual cost of a patient who experienced an asthma exacerbation (indicative of uncontrolled asthma) was more than 3.5 times the cost of those who did not experience an attack (£381 vs. £108)[7]. International studies have confirmed the high cost of managing exacerbations[8,9].

**Figure 1:**
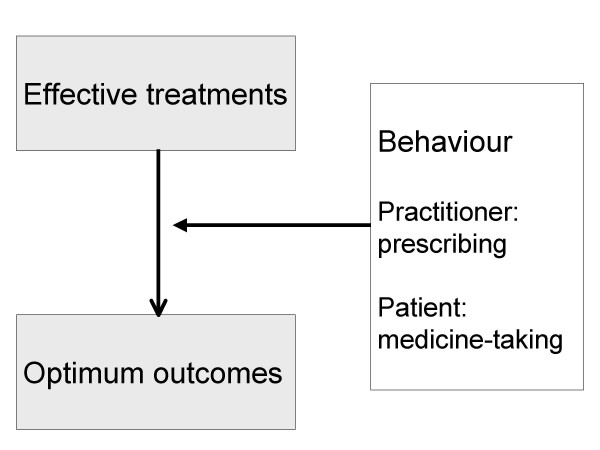
Patient and healthcare professional behaviour affects asthma control [10].

There are many possible reasons for poor control (Table 1). However, regardless of the underlying causes, the level of control achieved reflects the behaviour of both healthcare professionals and patients (Figure 1)[10]. Healthcare professionals need to conduct asthma reviews and take appropriate action if control is poor. Patients need to engage in self-management behaviours with optimal adherence to appropriate treatment. Differences in the perspectives of patients and healthcare professionals could affect their behaviours and consequently the achievement of asthma control. It may be possible for healthcare professionals to improve asthma control by achieving a greater understanding of the patient's perspective.

**Table 1:** Reasons for poor control

Co-morbidity (e.g. rhinitis, COPD)
Severe therapy-resistant disease
Ongoing exposure to triggers (e.g. occupational asthma, pets, mite etc)
Inadequate assessment
Misdiagnosis
Inadequate treatment
Ineffective delivery of treatment (e.g. poor inhaler technique)
Limited treatment effectiveness (e.g. smoking interfering with steroid actions)
Inadequate use of action plans
Low patient and physician expectations
Low adherence with agreed asthma therapy
Functional and psychological problems affecting willingness to use therapy
Over-reliance on complementary/alternative treatment
Not attending medical consultations
Patients do not perceive symptoms as indicative of poor control

These issues are of particular relevance in primary care, where the majority of patients with asthma are managed. An international panel of general practitioners, respiratory physicians, patient representatives and others with an interest in asthma control, under the auspices of the International Primary Care Respiratory Group (IPCRG), considered the evidence about patient perspectives and discussed the resulting implications. This report summarises the discussion.

## Discussion

Presentations by group members on different aspects of asthma control resulted in a wide ranging discussion that crystallised around three key questions.

1. What levels of asthma control are patients currently achieving?

2. What are the common causes of poor control?

3. What are the main patient-related determinants of asthma control?

Each of these questions is addressed, below.

### 1 – What levels of asthma control are patients currently achieving?

Large population-based studies, varying in methodology and funding, suggest that a substantial proportion of patients with asthma currently experience suboptimal levels of asthma control. The AIRE (Asthma Insights and Reality in Europe) study, involving over 2,800 people with asthma in France, Germany, Italy, Netherlands, Spain, Sweden and UK, found that asthma symptoms are part of everyday life for many patients[11]. More than half (56%) of the respondents (identified by telephone interviews of randomly selected households) suffered daytime symptoms in the last 4 weeks, and around one in three respondents experienced sleep disruption due to asthma at least once a week. Among the 753 children (<16 years) surveyed, 28% suffered night time symptoms in the previous month, with 61% needing to use their rescue medication.

Findings consistent with the AIRE study have been reported from the INSPIRE (INternational aSthma Patient Insight REsearch) study[12]. This study, conducted in eleven countries (Australia, Belgium, Canada, France, Germany, Italy, Netherlands, Spain, Sweden, UK, USA), included 3,415 adults with asthma treated with inhaled corticosteroids, recruited via their physicians and interviewed by telephone. Nearly three-quarters of the patients (74%) used a short-acting bronchodilator every day and half of all patients (51%) had at least one exacerbation requiring medical intervention in the past year. The mean number of asthma worsenings was 16 in those patients with uncontrolled asthma, compared with 6 in patients with well-controlled asthma.

### 2 – What are the common causes of poor control?

There are many reasons why asthma may be poorly controlled, both clinical and behavioural. Important clinical factors include the genetic characteristics of the individual, type of asthma (e.g. aspirin-sensitivity, neutrophilic activity), co-morbidity (e.g. dysfunctional breathing, allergic rhinitis)[13,14]. The behaviour of both clinicians and patients is also an important determinant of the level of asthma control achieved.

**Figure 2:**
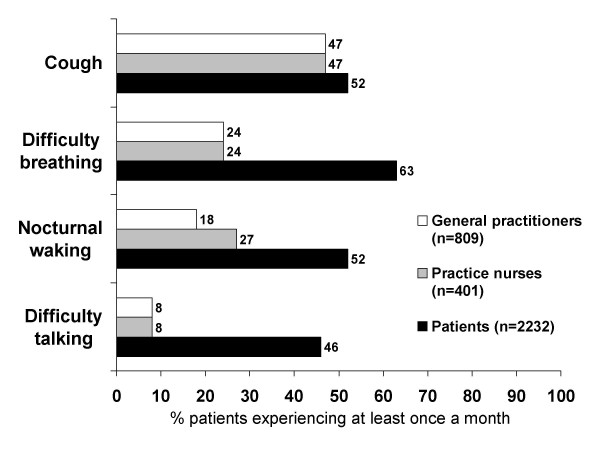
Healthcare professional and patient perspectives of the occurrence of asthma symptoms [16].

The behaviour of clinicians is vital in making an accurate diagnosis and prescribing the best treatment but also in carrying out appropriate review of progress and subsequent control[15]. Healthcare professionals may have limited awareness of symptom prevalence. In the AIR (Asthma in Real life) study, general practitioners substantially underestimated the prevalence of asthma symptoms (Figure 2)[16]. Furthermore, healthcare professionals may have difficulties estimating levels of asthma control[17,18]. Clearly, there is a need for healthcare professionals to appreciate the widespread occurrence of poor asthma control.

Patient behaviours are also key as the level of asthma control is influenced by adherence to treatment and other self-management behaviours[19] and smoking[20]. Patients' may also fail to consult their doctor. A UK survey found that 10% of asthmatic patients had seen no health professional about asthma in the previous 3 years[21].

### 3 – What are the main patient-related determinants of asthma control?

#### Patient expectations, aspirations and goals

Patients may unnecessarily accept symptoms, assuming that frequent symptoms, exacerbations and lifestyle limitations are an inevitable consequence of having asthma[22]. In the AIRE study, the majority of patients considered themselves to have controlled asthma, yet symptom levels showed control failing to reach the levels expected by management guidelines[11]. Patients may not realise that effective treatments are available. This was demonstrated in a study of 517 patients in the UK[23]. While 58% of patients reported that they were very satisfied with the standard of their asthma management, this fell to 33% after being shown the standards that patients can expect, as detailed in international guidelines. Such work implies that there is a need to raise patient expectations by increasing awareness of the quality of life that could be attained.

##### What level of control do patients want to achieve?

When asked about the things that they most dislike about asthma, patients most report symptoms such as cough, breathlessness, and lifestyle restrictions[16]. In a study of patients with asthma, 55% of respondents reported that they would find a written action plan helpful[23], though another study reported that 45% of patients neither had nor wanted regular asthma review[15]. Some of the goals that patients say they want are apparently contradictory (e.g. they may want few symptoms and no impact on activities, but do not want to take medication that could help achieve this). In real life, patients make choices between different attributes of the disease and its treatment, trading off one aspect for another – just as they may choose between consumer goods that offer different features at different costs. Discrete choice experiments allow integration of these different aspects in one measure by presenting patients with a choice of scenarios, each of which includes the key characteristics at different levels. Health technology assessment agencies, such as NICE, may use information from discrete choice experiments to understand the issues that matter to patients[24].

A discrete choice experiment showed that patients were willing to experience higher levels of wheeze and sleep disturbance to avoid cough and breathlessness [25]. However, patients who have not experienced an exacerbation may not rate avoidance of an attack as highly as those who have suffered one, possibly because of the pronounced impact of an exacerbation on quality of life[26]. Discrete choice experiments have also been used to assess patient preference for different treatment regimens[27] and for autonomy in decision-making in asthma management[28].

##### Patient goals and asthma control

Currently, asthma control is measured in ways defined by healthcare professionals (e.g. use of reliever medication, lung function, need for unscheduled healthcare). However, these standard methods use surrogate markers that do not seem to be necessarily relevant to the individual patient. In contrast, psychological treatments routinely use patient-defined goals, achievement of which can be seen by both patient and healthcare professional as markers of improvement. For example, an agoraphobic patient may set a goal of walking to the shop to buy a newspaper each day. Identifying and using patient goals has been shown to encourage patient involvement, which may lead to better adherence with therapy[29]. It is also consistent with the view that effective therapy is that which satisfies the patient's goals and expectations[30].

Few published studies have explored this issue in asthma with the exception of two recent studies. In the first study[31] in 329 adults with asthma, patients were asked to name up to three personal goals, achievement of which would indicate to them that their asthma treatment was effective. One year later, patients were asked to score the extent to which they had achieved their goals. Most patients (92%) were able to set three goals. Themes from patient-named goals were categorised qualitatively, subject to inter-rater reliability, with key words identified for each theme. Four main themes were identified: reducing activity limitation (named by 60% of participants), reducing asthma symptoms (named by 52% of patients), avoiding/reducing exacerbations (named by 46% of patients), and 38% wished to reduce their use of relief treatment. Within these themes, patients could name more specific behavioural goals (e.g. avoiding use of out-of-hours service, being able to play more sport). Patient-set goals appeared to be responsive to change. When asked to score the achievement of their goals at the end of the study, 49% of participants considered that they had achieved or partially achieved their own goals of treatment.

In the second study of 83 patients with exercise-induced asthma treated using montelukast or placebo, patients were also able to set their own goals[32]. The nature of these goals was very similar to that found previously, falling into four themes of reducing activity limitations, asthma symptoms, exacerbations and use of medication. These were more sensitive to change than the responses to the Royal College of Physicians' three questions[33].

#### Patient adherence to treatment and other aspects of self-management

Patients may not take the medications they have been prescribed, contributing to poor disease control. Regardless of age, gender and socioeconomic status of patients, and type and severity of disease, non-adherence rates of over 30% have consistently been noted across chronic illnesses[34] and with even higher rates of nonadherence to inhaled corticosteroids[35]. Non-adherence may be lower for more complex regimens, but significant non-adherence remains even when the frequency of dosing is reduced[36,37]. Furthermore, providing clear information – although essential – is not enough to guarantee adherence[38].

Nonadherence is best thought of as a variable behaviour, rather than a trait characteristic: most people are nonadherent some of the time. Nonadherence can have both intentional and unintentional causes. Unintentional nonadherence arises from capacity and resource limitations that prevent patients from implementing their decisions to follow treatment recommendations and involves individual constraints (e.g. poor inhaler technique, problems remembering doses etc) and aspects of their environment (e.g. problems of accessing prescriptions, cost, competing demands etc). Intentional nonadherence arises from the beliefs, attitudes and expectations that influence patients' motivation to begin and persist with the treatment regimen[37].

##### Patients' 'common-sense' beliefs about treatment and perceptions of asthma

Patients' self-management of their asthma is strongly influenced by their 'common-sense' beliefs about illness and treatment[39]. Patients do not blindly follow treatment advice even when it comes from trusted parishioners. Rather they evaluate whether the advice makes common sense in the light of their own understanding and beliefs about the illness and treatment [40]. Patients' adherence to medication is particularly influenced by the way in which they evaluate their personal *need *for medication relative to their *concerns *about potential negative effects of taking it. The utility of this simple necessity-concerns framework in explaining nonadherence has been shown in studies across a range of chronic illnesses [41-43], including asthma[44].

Patients are more likely to doubt the necessity of treatment if they do not perceive a good fit with their common-sense understanding of their illness and symptoms experiences relative to expectations[45,46]. A study of adherence to ICS in community-managed asthma patients in the UK showed that for many patients, the medical model of asthma as a chronic condition requiring daily preventer treatment was perceived to be at odds with their symptomatic experience of asthma as an episodic condition (e.g. my asthma isn't there when I don't have symptoms). These patients were more likely to doubt their personal need for daily ICS and were significantly less adherent[45]. Moreover, patients' concerns about potential adverse effects of medication become more salient when they doubt the necessity of treatment[40]. Patients' concerns about prescribed treatment extend beyond the experience of side effects to include more abstract worries arising from negative beliefs about pharmaceuticals such as the potential for dependence and long-term effects[47].

Mistrust of orthodox therapies may be one reason why many patients resort to unproven complimentary or alternative treatments for asthma. Surveys have shown high levels of use of such treatments by asthmatics in spite of a poor evidence base for efficacy[48].

Patients' beliefs about asthma and its treatment may be a hidden determinant of nonadherence when they are not volunteered in the consultation. If healthcare professionals are not aware of patient beliefs and hold different (sometimes opposing) beliefs about the nature of the illness and its appropriate treatment, then the consultation is unlikely to lead to successful outcomes. Where people lack information they may have inaccurate and uninformed views on the illness. They may also ignore missing information, devalue a treatment option, or make inferences based on the limited information they do have. Although patient beliefs govern their attitudes towards therapy, these beliefs are not fixed and can be changed through education and negotiation[49]. Finally, other psychological factors such as anxiety and depression may influence patient behaviour and asthma control[50]. Socioeconomic status[51] and ethnicity[52] are also important.

## Implications for practice

Three implications for health care professionals emerged from the debate on the patient's perspective of asthma control. The need

1. For simple tools to assess and monitor asthma control;

2. To identify the patient-related reasons for poor control;

3. To incorporate patient perspectives into the routine review of asthma in primary care

Each of these implications is discussed in turn, below.

### 1 – Assessing and monitoring asthma control

#### Severity vs. control

Management guidelines for asthma, as with many other diseases, include treatment algorithms based on severity of disease, as defined by the clinical features before treatment or by the treatment given[1,2]. Severity usually refers to the degree of underlying pathology. Deciding the severity of asthma is not always easy[53]. When respiratory specialists were shown a number of case studies and asked to assign the severity of the asthma for each case, there was considerable disagreement[54]. The inherent variability of asthma also presents problems for classification of severity[55]. Severe asthma, defined as persisting symptoms despite high levels of treatment, is likely to have a number of underlying reasons, including psychological and adherence factors[56].

Although the factors indicating control may be the same as those indicating severity (e.g. persistent symptoms, impaired lung function, high bronchodilator use, oral steroid use, unscheduled consultations, hospitalisations, life-threatening attacks), there is a difference in the two concepts. Patients with severe asthma can be well-controlled, while those with mild underlying disease can show signs of poorly controlled disease. Changing the management plan to one based on control and the goals of patients may show improved outcomes compared to a plan based on severity.

Hospital-based studies have suggested that outcomes may be better with treatment algorithms based on parameters that are more closely linked to control than usual symptom-based management protocols. There is a reduction in exacerbation rate, although a higher inhaled corticosteroid load received, in patients given treatments that optimise reduction in bronchial hyperreactivity, rather than treatment on the basis of symptoms[57]. Likewise, a treatment strategy based on normalisation of sputum eosinophil levels resulted in reduced exacerbations compared to standard management protocols, without increasing steroid exposure[58]. A randomised controlled trial conducted in New Zealand used a management strategy incorporating exhaled nitric oxide (NO) readings and achieved control that was at least as good as that obtained with a guideline-based approach but using a lower inhaled steroid dosage[59]. The GOAL study used a strategy based on the combined aims of treatment given in the GINA guidelines[1], and showed that the majority of patients treated with individually titrated doses of inhaled corticosteroids, either alone or in combination with long-acting beta_2_-agonist, could achieve and maintain control.

#### The need for simple valid and reliable measures of asthma control

In many chronic diseases, healthcare professionals have a philosophy of treating to achieve a predefined target level in a surrogate marker that indicates good control However, in asthma, there is currently no simple, clear, accepted target measure that healthcare professionals can aim to achieve, and that patients can use as a reliable indicator of treatment effectiveness. Instead, asthma control is currently implied in a number of ways (Table 2).

**Table 2:** Current methods used to imply level of asthma control

Measure	Comments
Symptoms	Day, night, exercise-induced
Lung function	% predicted, % variability
Healthcare resource use	Rescue medication, oral steroids, emergency consultations, hospitalisation
Bronchial hyperreactivity	Not suitable for routine clinical use
Biomarkers	Sputum eosinophils, exhaled NO
Health status	Numerous questionnaires available

Assessment of asthma has traditionally been based on parameters noted in management guidelines, such as lung function and symptoms. However, there is little correlation between commonly measured objective measurement of lung function using peak flow meters or spirometry and the level of symptoms or quality of life impairment perceived by patients[60], and less easily measured parameters such as lung hyper-inflation may show better correlations with symptoms such as breathlessness[61]. This may be due, in part, to other factors that influence perception of symptoms, such as concomitant anxiety, depression and socioeconomic status[51]. The demonstration of variability and reversibility of airflow limitation confirms asthma but the absence of these features at a given moment in time does not preclude the diagnosis.

A simple tool is required to assess asthma control accurately. The tool needs to be quick to use in primary care, where the majority of patients with asthma are managed by a range of healthcare professionals, in brief consultations. The ideal features of an asthma control tool are summarised in Table 3. Two approaches can be considered: monitoring of biometric markers of control and development of questionnaires to record patient-reported outcomes.

**Table 3:** Ideal features of a tool to assess asthma control

**C**onvenient to perform
**O**bjective measure of asthma control
Simple **N**umeric value
Give a clear **T**arget to guide treatment
**R**eliable, valid and responsive to changes in asthma control over time
Able to predict **O**utcomes
Complementary to **L**ung function tests

Biometric markers assess factors associated with the pathogenesis of asthma. The definition of asthma encompasses airways hyperresponsiveness and inflammation, which may both be more closely linked to asthma control than traditional measures of symptoms and lung function. Measurements of bronchial hyperreactivity have higher sensitivity and specificity for the diagnosis of asthma than measurements of diurnal variation in peak flow[62,63]. Exacerbations occur more frequently in patients with high bronchial reactivity, compared with those with reduced bronchial reactivity, though there is poor correlation between bronchial hyperreactivity and markers of inflammation. Bronchial hyperreactivity can be assessed by direct challenge (e.g. inhalation of histamine or methacholine) or indirectly (e.g. using exercise, or inhalation of hypertonic saline or mannitol). However, measurements of bronchial hyperreactivity are time consuming, and require appropriate equipment and healthcare professional expertise as well as patient cooperation, limiting the value of the approach as a practical measure of control. There is some interest in the use of mannitol BHR but this approach has yet to be fully evaluated in primary care.

Inflammation is central to the pathogenesis of asthma, with anti-inflammatory treatment forming the basis of asthma management. New technological developments enable non-invasive measurement of inflammation. Eosinophil count estimations in spontaneously produced or induced sputum can be measured as a marker of control, though the method requires appropriate expertise and laboratory support so is not currently suitable for use in routine primary care. Another surrogate measure of inflammation is exhaled nitric oxide (NO). NO is produced in low levels by airways epithelial and endothelial cells, but inflammatory cells contribute to greatly increased levels, explaining the observed correlation between NO-levels and eosinophilic inflammation[64]. Until recently, exhaled NO estimations necessitated the use of expensive monitoring equipment restricted to secondary care and research settings. However, technological advances have resulted in the development of inexpensive, handheld monitors to record exhaled NO, which are potentially available for use in primary care. This biometric method for assessment of control has some promise and is currently being evaluated.

##### Patient-based outcome measures

Patient-based outcome measures (Table 4)[65] may be useful to assess asthma control. A number of such tools have been developed that involve questioning the patient about outcomes achieved. Such tools may be generic or disease specific. Generic measures, such as the EQ-5D[66], are often easy to use, brief and acceptable to both patients and practitioners. They also capture the impact of comorbid conditions (which are common in patients with asthma) and allow comparison across patient groups and therapies. However, asthma-specific outcome measures are more sensitive than generic measures to disease-specific aspects of health-related quality of life and the effects of asthma treatments.

**Table 4:** Criteria for selecting patient-based outcome measures [65]

Criteria	Comments
Appropriateness	Match to the specific purpose and question to be addressed
Reliability	Reproducible and internally consistent
Validity	Measures what it purports to measure
Responsiveness	Sensitivity to changes of importance to patients
Precision	Number and accuracy of distinctions made by the instrument
Interpretability	How meaningful the scores are
Acceptability	How acceptable to the respondents
Feasibility	Effort, burden and disruption to staff and clinical care

There are a number of asthma-specific patient-based measures available that vary in characteristics, technical validity and ease of use. The Rule of Two™ consists of three items covering asthma symptoms and rescue medication use, each of which is answered with 'yes' (= 2 points) or 'no' (= 1 point) so that summing the answers gives a score ranging from 3 (= poor control) to 6 (= good control)[67]. Although some validation of the instrument has been carried out, there is no information on a minimal important difference and limited data on responsiveness. However, the instrument is quick and easy to use in consultations. The 30-second test is widely used in Canada, where it is recommended in management guidelines[68]. Although the six questions are easy and quick to answer with yes/no, responsiveness has not been determined. The Royal College of Physicians three questions are validated against other tools, widely used in the UK and recommended in management guidelines[33]. With the questions requiring simple yes/no answers, the method is quick and easy to use in clinical practice. Tools such as the Asthma Control Test™ (ACT)[69,70], and the Asthma Control Questionnaire™ (ACQ)[71,72] are also useful, and show good correlation with each other [53,73]. The ACT is shorter, requires no calculations and includes a question on the patient's view of control so gives a useful insight into the patient perspective. Both the ACT and ACQ are validated tools that are reliable and responsive to changes in asthma control over time, and provide a single numerical indication of control that has the potential to provide a target to drive management, analogous to that of a blood pressure measurement or a lipid measurement for management of hypertension or dyslipidaemia. Both measures have the potential to influence long-term asthma outcomes, raising expectations for asthma management and facilitating the achievement of asthma control.

### 2 – Identifying and addressing patient-related reasons for poor control

Identifying poor asthma control is the first step in improving it. The next step is to establish the reasons for poor control in individual patients. This should include an assessment of whether patient behaviours such as smoking or treatment nonadherence might be contributory factors. Patients may be reluctant to admit smoking or nonadherence if they believe that this will offend the clinician. However, asking adherence questions in a supportive manner, which sanctions nonadherence, can overcome this problem leading to more accurate reports[45].

It is also important to identify the specific reasons for nonadherence in individual patients. One of the reasons why previous interventions to improve adherence have met with limited success[74] is that they have taken a 'one-size fits all' approach, rather than individualising the approach to meet the specific needs of the patient[37]. Interventions to facilitate optimum adherence with asthma therapy are likely to be more effective if they are individualised and address perceptual barriers (e.g. patient beliefs and expectations) as well as practical barriers (e.g. regimen convenience, ability to use inhaler devices)[45].

Healthcare professionals can provide information about illness and treatment. However, unless the information given has an impact on patients' common-sense beliefs about the illness and treatment, it will not change patient behaviour. Healthcare professionals should ask about current illness and treatment beliefs. Although patient beliefs govern attitudes towards therapy, these beliefs are not fixed and can be changed through education and negotiation, leading to a better understanding of asthma that may promote more effective self-care behaviours[45]. In short, what people believe about their asthma may affect how they cope with it, and tailored education is the first stage. A three-step approach, covering perceptions and practicalities, has been suggested to facilitate optimal adherence to appropriately prescribed treatment[75]. This suggests that healthcare professionals should:

1) provide a common-sense rationale for the necessity of treatment that is consistent with the patient's common-sense model of asthma and their goals for asthma control

2) elicit and address specific concerns about treatment

3) prescribe a convenient treatment regimen tailored to address practical barriers to adherence (e.g. correct inhaler technique, use of combination inhalers to reduce inhaler load (if indicated)).

Identifying and addressing misplaced health beliefs (e.g. asthma is an episodic disease) may be helped by tailoring education to the patient's own needs and goals. Explicitly eliciting patient goals and using these as a basis for treatment and education may allow professionals to identify more effectively what is important to the patient and allow the patient to assess meaningful changes in their asthma. The importance of the patient in their own self-care is increasingly recognised, with development of initiatives to support self-care[76]. Teaching physicians to improve interactions with patients can result in greater ability to address patients' fears about asthma medication and improvement in asthma control[77].

### 3 – Incorporating patient perspectives into the routine review of asthma in primary care

The identification of non-adherence as a cause of poor control, and the factors contributing to poor adherence, has increased recognition of the need for individual patient-centred reviews. However, there are increasing resource constraints in primary care. Currently, asthma reviews are often not standardised in structure and data collection, are not comprehensive, fail to address the needs and expectations of patients, are ineffective at reducing morbidity and mortality, and are poorly attended.

The Minimal Asthma Assessment Tool (MAAT) is under development as a method to address some of these issues, and to help prioritise patients for primary care review by identifying poor asthma control and the causes of poor control for individual patients[78]. The MAAT consists of a brief 2-page questionnaire covering patient views about preventer inhalers, actual use and perceived side-effects, how asthma affects the individual, and issues likely to affect asthma control such as smoking and co-morbid rhinitis. An international study is planned to evaluate use of the MAAT. The development of effective tools to facilitate more efficient, patient-centred review of asthma in primary care is vital to improving asthma control and patient quality of life.

## Summary

It is possible to improve current levels of asthma control if healthcare professionals do four things:

1) use appropriate, patient-centred tools to assess control

2) identify the reasons for poor control in individual patients

3) work with patients to design individual treatment plans that address poor control and the causes of poor control, taking account of patient goals and aspirations

4) monitor outcomes and take appropriate action through regular review

## Competing interests

Most of the authors or their research teams have received honoraria at various times for their involvement in advisory panels or meetings, and/or funding for research projects from a number of companies marketing respiratory products. However, the authors declare that they have no competing interests that have affected their views expressed in this paper.

## Authors' contributions

All authors were present for the full duration of the discussion and contributed by making presentations and/or participating in the ensuing discussion. All authors have had the opportunity to read and amend draft versions of the manuscript.

## Pre-publication history

The pre-publication history for this paper can be accessed here:


